# Supplementary material for: The DNA-binding protein HTa from Thermoplasma acidophilum is an archaeal histone analog
Source: eLife. 2019 Nov 11;8:e52542. doi: 10.7554/eLife.52542 (PMC6877293; doi:10.7554/eLife.52542)
Supplement: Supplementary file 1. [file elife-52542-supp1.docx]

**Supplementary File 1. Representation of HU homologs across bacterial phyla.**

| **Phylum** | **Number of homologs detected** |
| --- | --- |
| Acidobacteria | 22 |
| Actinobacteria | 132 |
| Aquificae | 25 |
| Bacteroidetes | 142 |
| Caldiserica | 4 |
| Chlamydiae | 16 |
| Chlorobi | 4 |
| Chloroflexi | 25 |
| Chrysiogenetes | 4 |
| Cyanobacteria | 153 |
| Deferribacteres | 10 |
| Deinococcus–Thermus | 14 |
| Dictyoglomi | 2 |
| Elusimicrobia | 9 |
| Fibrobacteres | 8 |
| Firmicutes | 273 |
| Fusobacteria | 13 |
| Gemmatimonadetes | 10 |
| Lentisphaerae | 2 |
| Nitrospirae | 4 |
| Planctomycetes | 14 |
| Proteobacteria | 866 |
| Spirochaetes | 22 |
| Synergistetes | 7 |
| Tenericutes | 15 |
| Thermodesulfobacteria | 8 |
| Thermotogae | 35 |
| Verrucomicrobia | 47 |
