# Supplementary material for: The DNA-binding protein HTa from Thermoplasma acidophilum is an archaeal histone analog
Source: eLife. 2019 Nov 11;8:e52542. doi: 10.7554/eLife.52542 (PMC6877293; doi:10.7554/eLife.52542)
Supplement: Supplementary file 2. [file elife-52542-supp2.docx]

**Supplementary File 2. Examples of putative archaeal and eukaryotic homologs that likely represent contamination during genome assembly.**

| **Putative archaeal/eukaryotic sequence** | **Branches with…** | **Bootstrap support** |
| --- | --- | --- |
| *Pantholops hodgsonii*  [Tibetan antelope]  (XP_005980042.1) | *Asticcacaulis taihuensis* [Proteobacteria] (WP_090646987.1) | 88% |
| *Pantholops hodgsonii*  [Tibetan antelope]  (XP_005982097.1) | Ochrobactrum sp. PW1  [Proteobacteria]  (BBA74293.1) | 59% |
| *Aspergillus sclerotialis*  (RJE17645.1) | Methylobacterium sp. 4-46  [Proteobacteria]  (WP_012330955.1) | 49% |
| *Nephila clavipes*  [Golden silk orb-weaver]  (PRD22299.1) | Acinetobacter  [Proteobacteria]  (WP_086210128.1) | 96% |
| Thorarchaeota archaeon SMTZ1-83 (KXH76530.1) | *Filimonas lacunae*  [Bacteroidetes]  (SIT08387.1) | 30% |
| Lokiarchaeota archaeon CR 4 (OLS13623.1) | *Ktedonobacter racemifer*  [Cyanobacteria]  (WP_007906963.1) | 97% |
