# Supplementary material for: The DNA-binding protein HTa from Thermoplasma acidophilum is an archaeal histone analog
Source: eLife. 2019 Nov 11;8:e52542. doi: 10.7554/eLife.52542 (PMC6877293; doi:10.7554/eLife.52542)
Supplement: Supplementary file 3. [file elife-52542-supp3.docx]

**Supplementary File 3. Fourier filtering parameters.**

| **Species** | **Condition** | **Fragment size (bp)** | **PcVal** | **Thr (Zsc)** | **Number of peaks** |
| --- | --- | --- | --- | --- | --- |
| *T. acidophilum* | day1 | 40-65 | 0.02 | 0.25 | 11754 |
| *T. acidophilum* | day2 | 40-65 | 0.02 | 0.25 | 13925 |
| *T. acidophilum* | day3 | 40-65 | 0.02 | 0.25 | 14069 |
| *T. acidophilum* | day3.5 | 40-65 | 0.02 | 0.25 | 8441 |
| *T. acidophilum* | day1 | 70-100 | 0.0125 | 0.25 | 3359 |
| *T. acidophilum* | day2 | 70-100 | 0.0125 | 0.25 | 6887 |
| *T. acidophilum* | day3 | 70-100 | 0.0125 | 0.25 | 6862 |
| *T. acidophilum* | day3.5 | 70-100 | 0.0125 | 0.25 | 4472 |
| *M. fervidus* | Exponential phase | 60-70 | 0.02 | 0.25 | 5363 |
| *M. fervidus* | Exponential phase | 87-97 | 0.0125 | 0.25 | 4780 |
| *M. fervidus* | Exponential phase | 117-127 | 0.01 | 0.25 | 3694 |
| *T. kodakarensis* | Exponential phase | 55-65 | 0.02 | 0.25 | 8472 |
